# Survival according to the site of metastasis in triple-negative breast cancer patients: The Peruvian experience

**DOI:** 10.1371/journal.pone.0293833

**Published:** 2024-02-01

**Authors:** Luis Piedra-Delgado, Diego Chambergo-Michilot, Zaida Morante, Carlos Fairen, Fernando Jerves-Coello, Renato Luque-Benavides, Fresia Casas, Eduarda Bustamante, Cesar Razuri-Bustamante, J. Smith Torres-Roman, Hugo Fuentes, Henry Gomez, Alexis Narvaez-Rojas, Gabriel De la Cruz-Ku, Jhajaira Araujo

**Affiliations:** 1 Universidad Científica del Sur, Lima, Perú; 2 Departamento de Oncología Médica, Instituto Nacional de Enfermedades Neoplásicas, Lima, Perú; 3 Boston Medical Center, Boston, Massachusetts, United States of America; 4 Morristown Medical Center, Morristown, New Jersey, United States of America; 5 Universidad Peruana Cayetano Heredia, Lima, Peru; 6 Universidad Peruana de Ciencias Aplicadas, Lima, Peru; 7 Universidad Ricardo Palma, Lima, Peru; 8 Department of Surgical Oncology, Miller School of Medicine, University Of Miami, Miami, Florida, United States of America; 9 Escuela Profesional de Medicina Humana, Universidad Privada San Juan Bautista, Chorrillos, Lima, Peru; BMSCE: BMS College of Engineering, INDIA

## Abstract

**Background:**

Evidence regarding differences in survival associated with the site of metastasis in triple-negative breast cancer (TNBC) remains limited. Our aim was to analyze the overall survival (OS), distant relapse free survival (DRFS), and survival since the diagnosis of the relapse (MS), according to the side of metastasis.

**Methods:**

This was a retrospective study of TNBC patients with distant metastases at the Instituto Nacional de Enfermedades Neoplasicas (Lima, Peru) from 2000 to 2014. Prognostic factors were determined by multivariate Cox regression analysis.

**Results:**

In total, 309 patients were included. Regarding the type of metastasis, visceral metastasis accounted for 41% and the lung was the most frequent first site of metastasis (33.3%). With a median follow-up of 10.2 years, the 5-year DRFS and OS were 10% and 26%, respectively. N staging (N2-N3 vs. N0, HR = 1.49, 95%CI: 1.04–2.14), metastasis in visceral sites (vs. bone; HR = 1.55, 95%CI: 0.94–2.56), the central nervous system (vs. bone; HR = 1.88, 95% CI: 1.10–3.22), and multiple sites (vs. bone; HR = 2.55, 95%CI:1.53–4.25) were prognostic factors of OS whereas multiple metastasis (HR = 2.30, 95% CI: 1.42–3.72) was a predictor of MS. In terms of DRFS, there were no differences according to metastasis type or solid organ.

**Conclusion:**

TNBC patients with multiple metastasis and CNS metastasis have an increased risk of death compared to those with bone metastasis in terms of OS and MS.

## Introduction

Among the phenotypes of breast cancer, triple-negative breast cancer (TNBC) is characterized by the lack of expression of estrogen, progesterone, and human epidermal growth factor 2 (HER2) receptors [1]. TNBC is frequent in African American and Hispanic women [2], and accounts for 15–20% of all breast cancers [3].

This phenotype is well known for its aggressiveness, high heterogeneity, high percentage of metastasis to solid organs, and high relapse rates compared to other phenotypes. For instance, one quarter of patients with mild local disease present relapse with distant metastasis within the first three years after diagnosis [4]. Due to these characteristics in addition to the lack of targeted therapy, TNBC has a poor prognosis with a worse overall survival and breast cancer cause specific-survival [5].

It is estimated that 20–30% of early cases of breast cancer will develop metastasis and the site of distant recurrence is associated with the breast cancer subtype [6]. TNBC has a higher rate of metastasis in lung, bone and brain, as well as multiple metastasis [7, 8]. Although the literature has shown that TNBC generally has a lower OS and relapse-free survival (RFS) than other phenotypes, data on survival according to the site of distant metastasis is still limited.

Hence, our aim was to analyze the OS and distant relapse free survival (DRFS) according to the site of metastasis in women with TNBC. Moreover, as a secondary objective, we analyzed the survival since the diagnosis of the relapse (MS) in TNBC patients.

## Materials and methods

### Study design and patient population

This was retrospective cohort study. We included and reviewed medical records from TNBC patients diagnosed and treated at the “Instituto Nacional de Enfermedades Neoplasicas” (Lima, Peru) from 2000 to 2014. The follow-up was until February 2020. The data were collected from December 2017 to March 2020. Inclusion criteria were as follows: age 18 years and older, breast cancer patients with confirmed a triple negative phenotype, stages I-III at diagnosis, and patients with distant relapse. The study included all the patients who met the inclusion and exclusion criteria. Cases lost to follow-up were excluded.

TNBC were identified by immunohistochemistry as estrogen receptor negative or <1%, progesterone receptor negative or <1%, and HER-2 negative or <1%. Patients with inconclusive staining for HER2 underwent fluorescence in situ hybridization; negativity was considered with a result < 2. Tumor stage was classified according to the 8^th^ edition of the American Joint Committee on Cancer (AJCC) [9]. The body mass index was classified as underweight (<18.5 kg/m^2^), normal (18.5–24.9 kg/m^2^), overweight (25.0–29.9 kg/m^2^) and, obese (≥30 kg/m^2^).

The type of metastasis was classified as visceral, central nervous system (CNS), bone, and multiple sites. Visceral metastasis was defined as the spread of cancer cells to adjacent or distant organ sites inside the thorax or abdominal cavity; whereas CNS metastasis included dura, brain parenchyma, and leptomeninges; while, bone metastasis referred to disease extension to bone. Multiple metastasis involved disease extension to multiple sites, such as bone and one solid organ or multiple solid organs. Later metastases were not considered in the initial classification.

### Follow-up

OS was considered as from diagnosis of the primary tumor until death or the end of the study. DRFS was considered as from the date of the beginning of any type of treatment until the diagnosis of the first distant metastasis, death, or the end of the study. MS was considered from the diagnosis of distant metastasis to the death or the end of study.

Distant relapse of breast cancer was identified by computed tomography, magnetic resonance imaging, bone scintigraphy, or positron emission tomography.

### Statistical analysis

For quantitative variables we used means and standard deviation (SD) or median with the interquartile range according to the distribution of data, while frequencies and percentages were estimated for qualitative variables. For univariate analysis, the Student’s t or Mann-Whitney U test (according to the distribution) was used for quantitative variables, while the chi-square test was used for qualitative variables. Survival rates were calculated using the Kaplan-Meier method, while differences between types of metastases were assessed by the Log rank test. Missing data were reported but were not included in the statistical analysis. Prognostic factors and their hazard ratios (HR) were estimated with multivariate Cox regression analysis. To address any potential sources of bias, we included all the population available in our analyses. All the analysis had a 95% of confidence interval (95% CI), and a p value <0.05 was considered statistically significant. All the analyses were performed with the Statistical Package for the Social Sciences (SPSS) v.24 and Stata 17 (College Station, TX: StataCorp LLC).

## Results

Ot he 534 TNBC patients presenting recurrence, 390 with distant metastasis (73.03%) were included in the study. The mean age was 48.3 years (SD: 11.9), 98 (25.2%) patients were obese and half were premenopausal (51.0%). The majority of patients had stage III TNBC at diagnosis (78.7%), followed by women with stage II (19.0%), and there were 7 cases of bilateral breast cancer (Table 1**).** The majority of patients (82.2%) underwent surgery, 58.5% received neoadjuvant chemotherapy (NAC), 41.5% received adjuvant chemotherapy (ACT), and 64.4% received radiotherapy.

**Table 1:** Clinical and pathological characteristics of patients with triple negative breast cancer presenting distant relapse.

Characteristics	Total N = 390	
	N	%
Age (years)		
≤35	53	13.6
36–49	170	43.6
≥50	167	42.8
BMI (kg/m^2^)		
18.5–24.99	155	39.7
25–29.99	137	35.1
≥30	98	25.2
Menopausal status		
Pre-menopausal	199	51.0
Post-menopausal	191	49.0
FHBOC		
No	343	87.9
Yes	47	12.1
Laterality		
Right	189	48.5
Left	194	49.7
Bilateral	7	1.8
T		
T1	10	2.7
T2	97	26.1
T3	65	17.5
T4	200	53.8
Unknown	18	
N		
N0	83	21.9
N1	166	43.8
N2	82	21.6
N3	48	12.7
Unknown	11	
AJCC Stage		
I	9	2.3
II	74	19.0
III	307	78.7
Histological Grade		
I	2	0.6
II	72	21.3
III	264	78.1
Unknown	52	
Histological subtype		
Ductal	371	95.6
Lobular	7	1.8
Medullar	3	0.8
Metaplastic	2	0.5
Epidermoid	2	0.5
Apocrine	1	0.3
Papillary	1	0.3
Inflammatory	1	0.3
Unknown	2	

BMI, body mass index; FHBOC, Family history of breast and/or ovarian cancer, AJCC, American Joint Committee on Cancer.

The lung was the most frequent first site of metastasis (33.3%), followed by multiple metastasis (29.2%), brain (18.5%), bone (9.7%), liver (6.7%), and leptomeningeal metastasis (1.5%). Regarding the type of metastasis, visceral metastasis accounted for 41% (Table 2).

**Table 2:** Surgical characteristics, treatment, and outcomes of patients with triple negative breast cancer with distant relapse.

Characteristics	Total N = 390	
	N	%
Type of surgery		
Conservative	65	16.7
Mastectomy	258	66.2
Not surgical intervention	67	17.2
Type of chemotherapy		
Neoadjuvant	228	58.5
Adjuvant	162	41.5
Adjuvant radiotherapy		
No	139	35.6
Yes	251	64.4
Loco-regional relapse		
No	249	63.8
Yes	141	36.2
First place of metastasis		
Lung	130	33.3
Brain	72	18.5
Bone	38	9.7
Liver	26	6.7
Leptomeningeal	6	1.5
Ovarian	3	0.8
Renal	1	0.3
Multiple sites	114	29.2
Site of distant metastasis		
Lung	183	46.9
Brain	144	36.9
Bone	111	28.5
Liver	93	23.8
Type of metastasis		
Bone	38	9.7
Visceral	160	41.0
Central Nervous System	78	20
Multiple	114	29.2
Death	348	89.2

Compared to visceral, CNS, or multiple metastasis; bone metastasis was more frequent in patients with a family history of breast or ovarian cancer (P = 0.02). N staging significantly differed among the four groups, with patients with multiple metastasis sites presenting similar lymph node involvement to that of patients with visceral and CNS metastasis, while patients with bone metastasis had a higher number of affected lymph nodes (P = 0.02). Finally, death was more frequent in the group with multiple metastasis sites (97.4%) compared to the groups of CNS (88.5%),visceral (85.6), and bone metastasis (81.6%) (P<0.01) (Table 3).

**Table 3:** Sociodemographic, clinical, pathological, surgical characteristics and outcomes of patients with triple negative breast cancer with distant relapse according to the type of metastasis.

Characteristics	Type of metastasis				Total N = 390	p value
	Visceral N = 160	Central Nervous System N = 78	Bone N = 38	Multiple sites N = 114		
Age Mean (standard deviation)	48.6 (12.6)	47.8 (11.5)	47.7 (9.9)	48.3 (11.9)	48.3 (11.9)	0.94 ^a^
BMI (kg/m^2^) Mean (standard deviation)	27.9 (4.9)	27.8 (4.2)	27.9 (5.4)	27.0 (4.1)	27.7 (4.6)	0.20 ^a^
Menopausal Status						
Pre-menopausal	82 (51.3)	39 (50.0)	20 (52.6)	58 (51.3)	199 (51.0)	0.99 ^b^
Post-menopausal	78 (48.8)	39 (50.0)	18 (47.4)	56 (49.1)	191 (49.0)	
FHBOC						
No	145 (90.6)	71 (91.0)	36 (94.7)	91 (79.8)	343 (87.9)	0.02 ^b^
Yes	15 (9.4)	7 (9.0)	2 (5.3)	23 (20.2)	47 (12.1)	
T						
T1-T2	44 (28.4)	18 (24.7)	11 (31.4)	34 (31.2)	107 (28.8)	0.41 ^b^
T3	21 (13.5)	19 (26.0)	6 (17.1)	19 (17.4)	65 (17.5)	
T4	90 (58.1)	36 (49.3)	18 (51.4)	56 (51.4)	200 (53.8)	
Unknown	5	5	3	5	18	
N						
N0	39 (25.0)	13 (17.1)	5 (13.9)	26 (23.4)	83 (21.9)	0.02 ^b^
N1	74 (47.4)	29 (38.2)	12 (33.3)	51 (45.9)	166 (43.8)	
N2	28 (17.9)	21 (27.6)	8 (22.2)	25 (22.5)	82 (21.6)	
N3	15 (9.6)	13 (17.1)	11 (30.6)	9 (8.1)	48 (12.7)	
Unknown	4	2	2	3	11	
Laterality						
Right	89 (56.3)	31 (40.3)	20 (55.6)	49 (43.8)	189 (49.3)	0.06 ^b^
Left	69 (43.7)	46 (59.7)	16 (44.4)	63 (56.3)	194 (50.7)	
AJCC Stage						
I-II	41 (25.6)	12 (15.4)	6 (15.8)	24 (21.1)	83 (21.3)	0.25 ^b^
III	119 (74.4)	66 (84.6)	32 (84.2)	90 (78.9)	307 (78.7)	
Histological Grade						
I—II	23 (16.4)	19 (26.8)	8 (27.6)	24 (24.5)	74 (21.9)	0.23 ^b^
III	117 (83.6)	52 (73.2)	21 (72.4)	74 (75.5)	264 (78.1)	
Unknown	20	7	9	16	52	
Chemotherapy type						0.53 ^b^
Neoadjuvant	93 (58.1)	51 (65.4)	21 (55.3)	63 (55.3)	228 (58.5)	
Adjuvant	67 (41.9)	27 (34.6)	17 (44.7)	51 (44.7)	162 (41.5)	
Type of surgery						
Conservative	28 (20.9)	13 (20.0)	6 (20.0)	18 (19.1)	65 (20.1)	0.99 ^b^
Mastectomy	106 (79.1)	52 (80.0)	24 (80.0)	76 (80.9)	258 (79.9)	
Radiotherapy						
No	61 (38.1)	24 (30.8)	13 (34.2)	41 (36.0)	139 (35.6)	0.74 ^b^
Yes	99 (61.9)	54 (69.2)	25 (65.8)	73 (64.0)	251 (64.4)	
Death						
No	23 (14.4)	9 (11.5)	7 (18.4)	3 (2.6_	42 (10.8)	<0.01 ^b^
Yes	137 (85.6)	69 (88.5)	31 (81.6)	111 (97.4)	348 (89.2)	

BMI, body mass index; FHBOC, Family history of breast and/or ovarian cancer, AJCC, American Joint Committee on Cancer.

^a^ p-value calculated with ANOVA test

^b^ p-value calculated with chi-square

**Fig 1:**
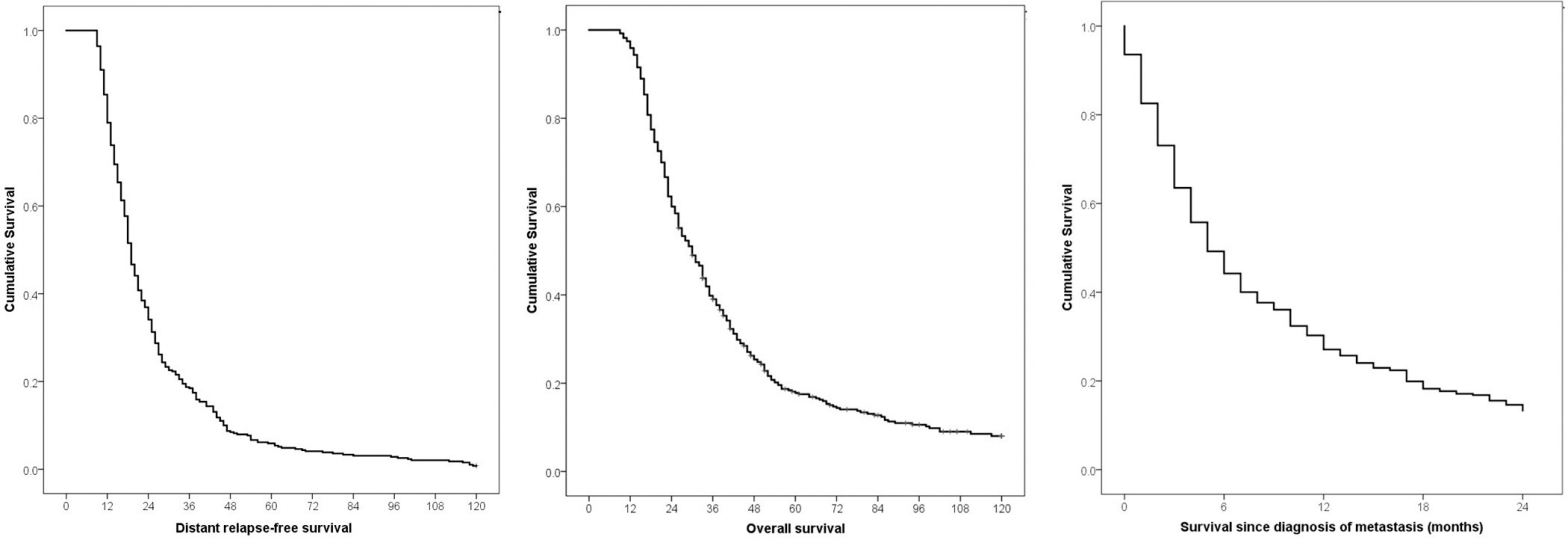
Relapse free survival (A), overall survival (B), and survival since diagnosis of first metastasis (C) of patients with triple negative breast cancer with distant metastasis.

The study population was followed for a median of 10.2 years. The DRFS at 3 and 5 years was 30% and 10%, respectively (Fig 1), **while** the OS was 44% and 26%, respectively (Fig 1).

**Fig 2:**
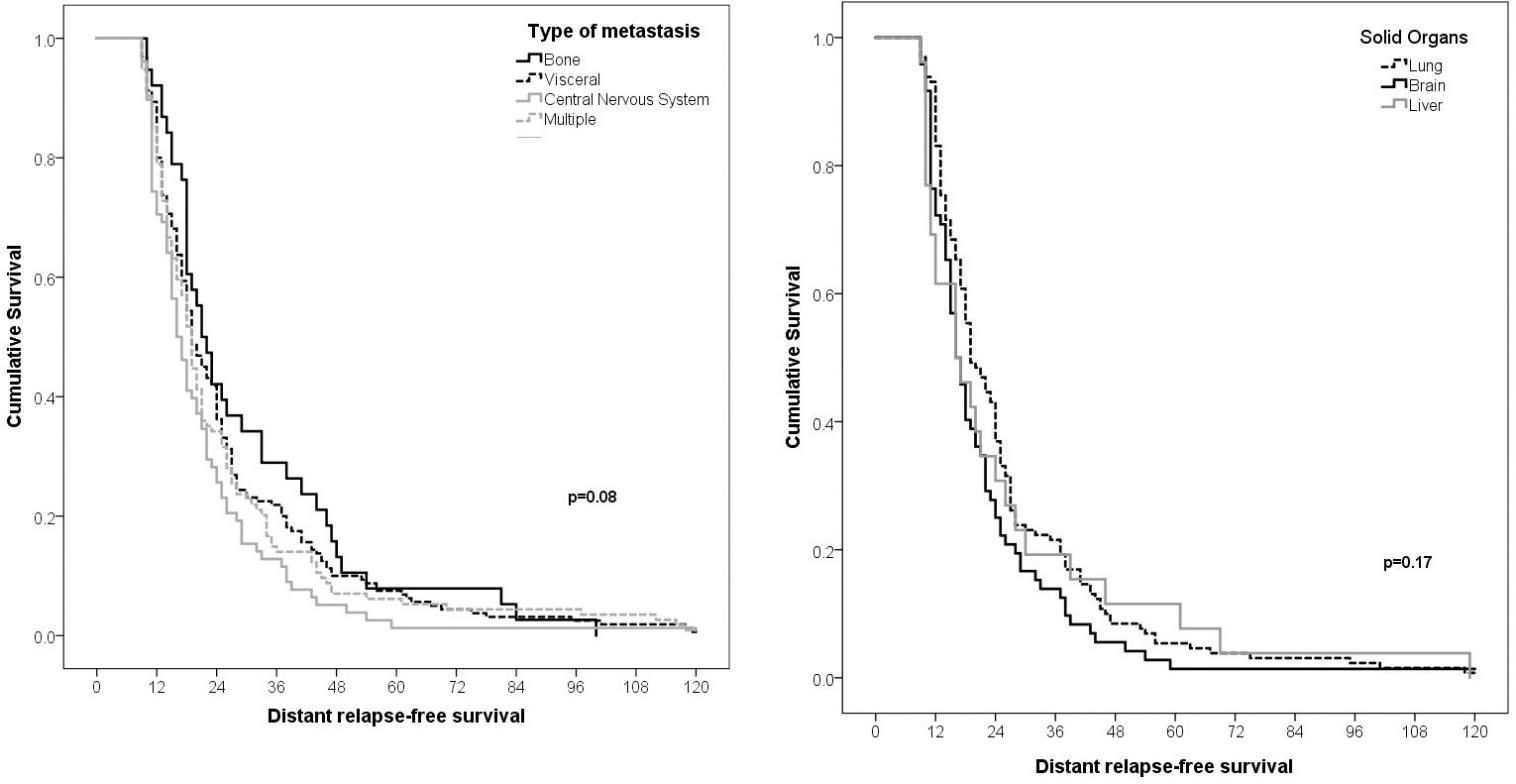
Comparison of relapse-free survival of patients with triple negative breast cancer from the time of diagnosis of primary cancer to relapse according to the type of metastasis (A) and solid organs (B).

**Fig 3:**
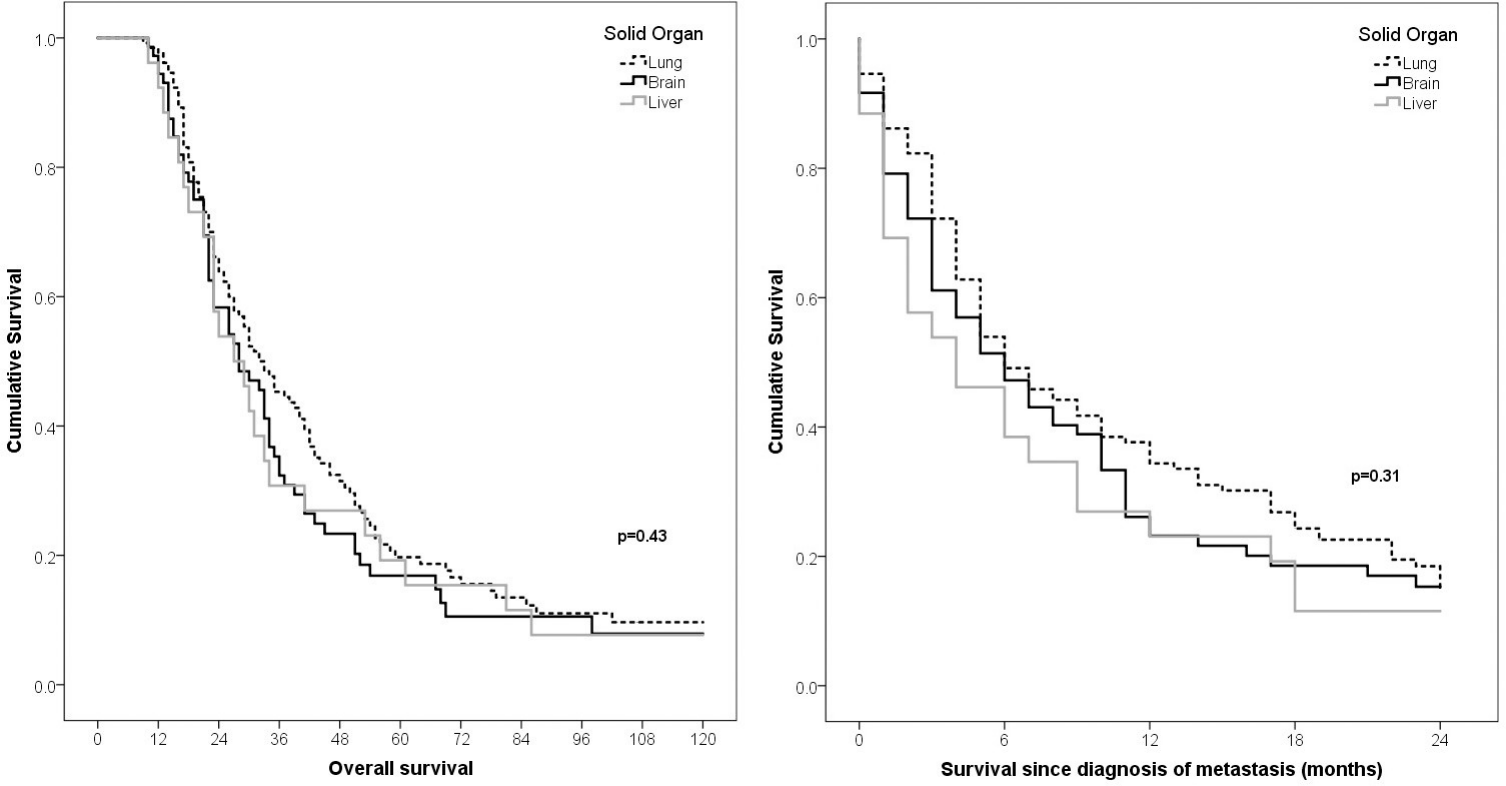
Comparison of overall survival of patients with triple negative breast cancer with distant relapse (A) and metastasis survival since the diagnosis of first metastasis (B) according to solid organ metastasis.

**Fig 4:**
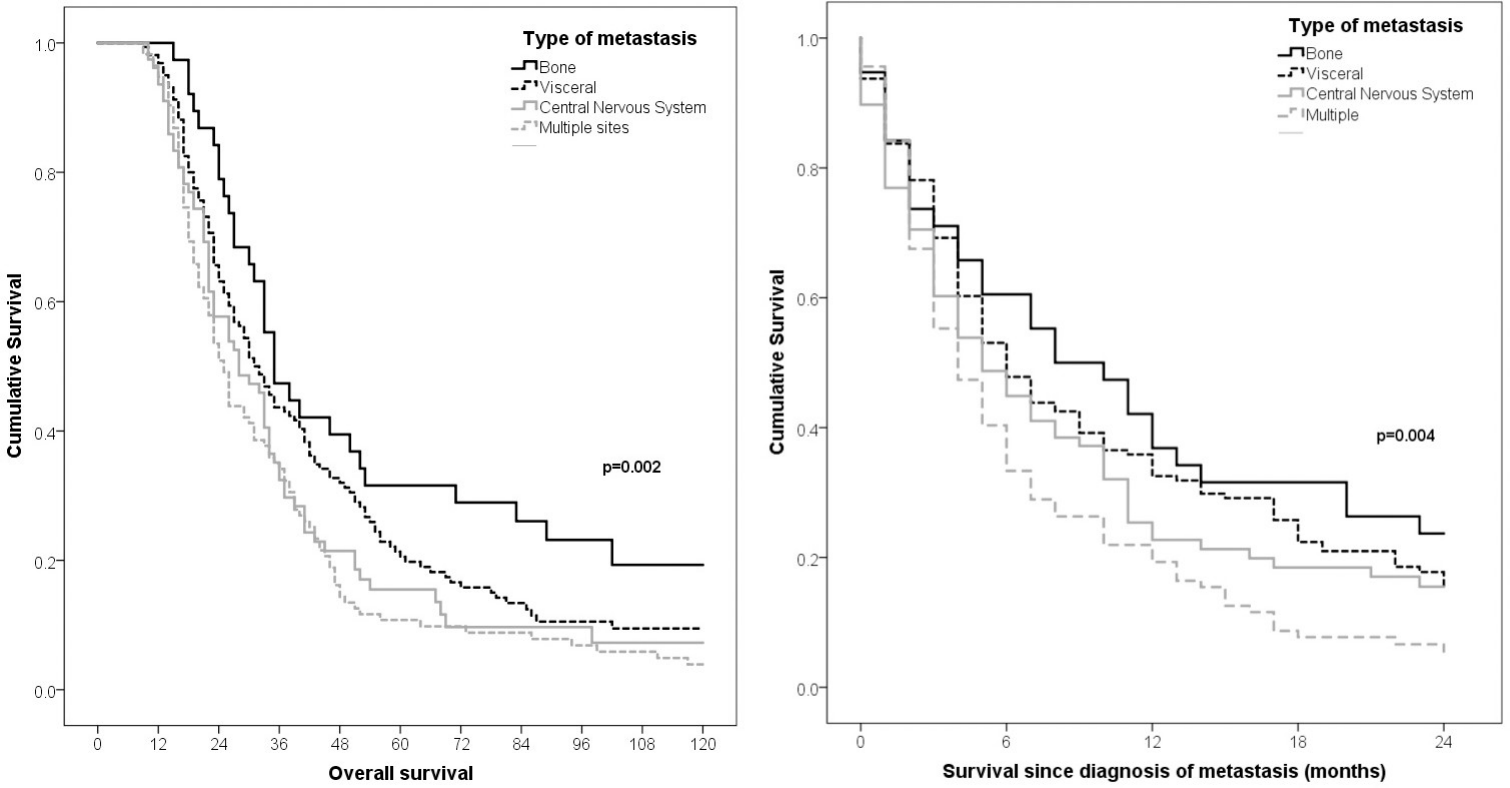
Comparison of overall survival of patients with triple negative breast cancer with distant relapse according to the type of metastasis of the primary cancer (A) and metastasis survival since the diagnosis of first metastasis (B).

There were no differences when DRFS was assessed according to the type of metastasis (p = 0.08). Similarly, there were no differences when comparing the survival of patients with metastasis with lung, brain, or liver (p = 0.17) (Figs 2 and 3). However, the OS and MS rates were lower in patients with multiple sites of metastasis, followed by visceral, CNS, and bone metastasis at 3 years (34%, 44%, 62%, and 47%) and at 6 months (33%, 48%, 45%, and 61%), respectively (Fig 4).

Cox regression multivariate analysis showed the predictors of a worse OS: N staging (N2-N3 vs. N0, HR: 1.49; 95% CI: 1.04–2.14), metastasis to CNS (vs. bone; HR: 1.88; 95% CI: 1.10–3.22), and multiple metastases (vs. bone; HR: 2.55; 95% CI: 1.53–4.24) (Table 4). Moreover, the only predictor of a worse MS was the presence of multiple metastasis (HR: 2.30; 95% CI:1.42–3.72) (Table 5).

**Table 4:** Cox regression analysis for the overall survival of patients with triple-negative breast cancer with distant metastasis with tumor stage I to III.

Characteristics	Overall survival					
	Univariate analysis			Multivariate analysis		
	HR	95% CI	P value	HR	95% CI	p value
Age	0.99	0.98–1.00	0.17	0.99	0.98–1.01	0.42
FHBOC						
No	1.00			1.00		
Yes	0.69	0.51–0.95	0.02	0.74	0.48–1.14	0.17
T						
T1-T2	1.00			1.00		
T3	1.99	1.46–2.71	<0.01	1.21	0.78–1.88	0.40
T4	2.00	1.59–2.50	<0.01	1.15	0.80–1.66	0.46
N						
N0	1.00			1.00		
N1	1.48	1.15–1.91	<0.01	1.22	0.86–1.75	0.27
N2-N3	1.75	1.35–2.28	<0.01	1.49	1.04–2.14	0.03
Histological grade						
I-II	1.00			1.00		
III	0.96	0.75–1.22	0.74	1.13	0.81–1.57	0.49
Neoadjuvant chemotherapy						
No	1.00			1.00		
Yes	2.21	1.83–2.70	<0.01	1.27	0.86–1.87	0.24
Type of surgery						
Conservative	1.00			1.00		
Mastectomy	1.76	1.35–2.29	<0.01	1.29	0.89–1.87	0.17
Adjuvant chemotherapy						
No	1.00			1.00		
Yes	0.56	0.46–0.67	<0.01	0.82	0.59–1.16	0.26
Radiotherapy						
No	1.00			1.00		
Yes	1.04	0.86–1.27	0.67	1.23	0.91–1.66	0.19
Type of metastasis						
Bone	1.00			1.00		
Visceral	1.40	0.95–2.08	0.09	1.55	0.94–2.56	0.08
Central Nervous System	1.70	1.11–2.60	0.02	1.88	1.10–3.22	0.02
Multiple sites	1.99	1.34–2.98	<0.01	2.55	1.53–4.25	<0.01

FHBOC, Family history of breast and/or ovarian cancer; HR: hazard ratio; CI: confidence interval.

**Table 5:** Cox regression analysis of the survival of patients with triple-negative breast cancer with distant metastasis with tumor stage I to III following the diagnosis of metastasis.

Characteristics	Survival since the diagnosis of the relapse					
	Univariate analysis			Multivariate analysis		
	HR	95% CI	P value	HR	95% CI	p value
Age	0.99	0.99–1.01	0.84	1.01	0.99–1.02	0.08
FHBOC						
No	1.00			1.00		
Yes	0.75	0.54–1.04	0.09	0.76	0.52–1.11	0.15
T						
T1-T2	1.00			1.00		
T3	1.35	0.96–1.86	0.07	1.40	0.97–2.03	0.08
T4	1.13	0.89–1.44	0.31	1.24	0.94–1.64	0.13
N						
N0	1.00			1.00		
N1	1.10	0.83–1.44	0.51	1.06	0.77–1.46	0.74
N2-N3	1.16	0.87–1.53	0.32	1.17	0.84–1.63	0.36
Histological grade						
I-II	1.00			1.00		
III	0.89	0.69–1.16	0.40	0.82	0.62–1.10	0.19
Type of metastasis						
Bone	1.00			1.00		
Visceral	1.32	0.89	0.17	1.47	0.92–2.36	0.11
Central Nervous System	1.46	0.95–2.23	0.08	1.66	1.01–2.73	0.05
Multiple sites	1.95	1.30–2.92	<0.01	2.30	1.42–3.72	<0.01

FHBOC: Family history of breast and/or ovarian cancer; HR: hazard ratio; CI: confidence interval.

## Discussion

We evaluated the association between the site of metastasis site and clinical outcomes in patients with TNBC. The results showed that N staging, CNS metastasis to and multiple metastasis were significant prognostic factors for a worse OS, and multiple metastasis was the only predictor of a worse MS in patients with TNBC and distant metastases.

With regards to the demographic characteristics of TNBC patients, our results are consistent with previous literature. The mean age of our patients was 48 years old, being similar to previous studies describing the diagnosis of TNBC in patients no older than 50 years old. Indeed, TNBC is more frequent in younger women compared to other phenotypes of breast cancer [10, 11]. Hence, TNBC is mainly presented in pre-menopausal women, although this factor has not been associated with an impact on survival outcomes [12, 13].

The majority of our patients were diagnosed in stage III (78.7%). TNBC patients are less likely to be diagnosed in a screening mammography, leading to diagnosis being made at a more advance stage [11, 14]. Moreover, In Latin America most cases are diagnosed in advanced stages due to the serious problem of limited access to breast cancer screenings in these countries [15, 16] Asad et al. found that the stage at diagnosis was the most significant factor for the rapid development of relapse of TNBC; with 55% of patients with stage III TNBC at diagnosis developing relapse, demonstrating a 15-fold higher probability of relapse compared to women with stage I [17]. The majority of our patients (78.1%) have a histologic grade III. In fact, almost all TNBC tumours are grade III invasive ductal adenocarcinomas [18, 19]. Cheung et al. reported a correlation between histological grade and the expression of the *snail2* protein, which is linked to transformation of tumor cells to the mesenchymal phenotype, thereby enabling metastasis and suggesting an association between histological grade and TNBC metastasis [20].

In spite of the fact that all the patients included in this study received either NAC, ACT, and/or radiotherapy, all of them developed relapse. In accordance with a prior cohort study about recurrence of TNBC; results showed that despite the majority of patients after undergoing adjuvant therapy, a dramatically high rate of recurrence was still observed [21]. A possible explanation of this outcome may be found in the study by Kim et al., which showed that breast cancer cells can develop resistance or even have pre-existing resistance to chemotherapy [22]. However, a meta-analysis by Yang et al. found that adjuvant chemotherapy reduced the rate of recurrence for T1a/bN0 TNBC [23]. Albeit promising, these results cannot be extrapolated to our investigation as the patients in the aforementioned study were all T1 whereas more than the 95% of our patients were T2 and above. Although the current NCCN guidelines are well established for each tumour size and lymph node invasion [24], information on the efficacy of chemotherapy and reduction of recurrence in advanced TNBC stages is still limited, and therefore additional studies are needed to elucidate the true role of the chemotherapy in TNBC relapse.

Predominant distant metastasis is a documented finding in TNBC, Dent et al. found that women with TNBC had an increased likelihood of distant recurrence within five years after diagnosis [25]. In fact, research suggests that the high risk and predominance of distant recurrence is in part caused by the excess of visceral and CNS metastases, but diminished bone recurrence seen in patients in the first five years after diagnosis [26, 27]. Some explanations for the tendency of TNBC to present visceral and CNS metastasis include the association between metastasis to certain organs and the expression of specific genes by the tumour [28]. Hicks et al. described that breast cancer patients that developed brain metastasis were likely to express cytokeratin 5 and 6 markers [29]. The expression of cytokeratin 5 is linked to the phenotype of cells undergoing the epithelial-mesenchymal transition, which is used by the tumour to gain migratory and invasive properties, thereby causing metastasis [30–32].

We found that lung metastasis was the most common site of metastasis similar to what other authors described, and was also the most common first site of invasion, followed by multiple metastasis and brain [33–38]. However, it has been proposed that the genes present in TNBC comprise the signature of lung metastasis, being a possible explanation of this phenomenon [35, 38–40]. The signature genes identified include the EGFR ligand epiregulin, COX2 and matrix metalloproteinases 1 and 2 [41]. Expression of these genes is important for motility but also allows angiogenesis, and intravasation of tumor cells into the circulation, which may be a factor of lung extravasation via the lung capillaries [40, 42]. Epiregulin and EGFR are of particular interest with several studies having found a strong correlation with their expression in TNBC [43–48]. The expression of EGFR has been reported as being inversely related to hormone receptor expression [43, 49, 50], suggesting that lung metastasis may be an intrinsic feature of TNBC.

Our results showed that the DRFS was 30% and 10% at three and five years, respectively. Due to the lack of specific targets, treatment with standard agents leaves patients prone to systemic and local relapse [48]. Cho et al. proposed that high expression of EGFR as well mutations of BRCA1 are linked to a worse RFS in TNBC [51]. Further evidence for EGFR as a marker for unfavourable prognosis is that expression of the aforementioned gene is linked to resistance to chemotherapy and radiation treatment, inherently forewarning poor survival [49, 50, 52].

We found no significant difference in disease-free metastasis, regardless of the organ invaded. This might be explained by the overall inefficiency of the available treatments. Treatment options are limited to standard chemotherapy and there is a current lack of targeted therapies. Despite an apparent responsiveness to these agents, the prognosis remains poor due to the higher likelihood of visceral relapse; a phenomenon referred to as the TNBC paradox [53–55]. This statement is demonstrated in the study by Fornier et al. [56]; who reported rapid progression following multiple lines of chemotherapy.

Regarding the stratification of risk factors according to the sites of metastasis, we found that a family history of breast or ovarian cancer was associated with higher rates of metastasis to multiple sites. Family history as a risk factor is mainly due to the association with BRCA1 mutations [57], which have been identified as contributors to the metastatic and aggressive nature of tumor cells [58]. Fasching et al. [57] reported that brain metastasis was frequently seen in patients with BRCA1 mutations whereas Song et al. [59] found that carriers of this mutation frequently experience lung and lymph node metastasis. On the same hand, N1 tumor staging was associated with metastasis to visceral, CNS, and multiple sites. Similar to our results, Lin et al. described that lower lymph node involvement (N1) was associated with a greater risk of metastasis to solid organs such as the brain and lung [60]. Finally, the association between death and metastasis to CNS and multiple sites showed the greatest significance. As shown in Table 3, multiple metastasis was related to death in 97.4% of cases whereas CNS and visceral metastasis to were associated with death in 88.5% and 85.6% of cases, respectively.

The average OS in the present study was lower than that described in a previous study (26% at 5 years) [61]. It has been reported, that the outcomes of Latin American breast cancer patients are worse compared to patients from developed countries. These poor outcomes are mainly associated with socioeconomic factors that not only limit access to screening, favoring late diagnosis, but also cause deficient access to specialised cancer care [15, 16]. This should be one of the major priorities for intervention in our setting to achieve better short and long term outcomes in our population.

When comparing the different sites of metastases, the lowest OS rates were found in patients with multiple metastases, followed by CNS, visceral, and bone. Supporting the latter, Wang et al. and other authors found that patients with metastasis to visceral, CNS and multiple sites had a lower OS, whilst those with bone metastasis had the highest OS amongst [61–63]. This last finding, together with the fact that TNBC tends to invade visceral sites more frequently than bone [64], is a framework to establish that worse prognosis and unfavorable OS in metastatic TNBC, are related to the excess risk and high incidence of visceral metastasis [27]. Therefore, the invasion of multiple viscera burdens carries the worst prognosis due to life threatening and life diminishing clinical presentations [63, 65].

We found that metastasis to CNS, N staging and multiple metastasis were prognostic factors for OS in TNBC. It was expected that a well-documented prognostic factor such as lymph node status [66–68], would be significant in multivariate analysis. In a study conducted using the SEER database, Gao et al. found that TNBC patients with distant metastasis have worse OS if they were IV stage, uninsured, did not undergo surgery and chemotherapy, and had metastasis in brain and liver [69]. However, to our knowledge this is the first instance in which multiple metastasis has been identified as such a significant prognostic factor for OS and MS in metastatic TNBC.

## Limitations

This was a retrospective study and thus, the associations among the factors analysed can be assured but not causation. Some patients were lost during follow-up, and were thereby excluded from the study, limiting maximum data collection and analysis. Moreover, test results for *BRCA* gene mutation was not available in the majority of our patients due to non-coverage by national insurance. In addition, although we included neoadjuvant and adjuvant chemotherapy, and radiotherapy in our analyses, we did not have further therapeutic information such as the chemotherapy regimen This information should be included in future studies due to the possible effects on the outcomes of this specific population.

Although the data used belonged to the main Peruvian Cancer Institute, patients from private centres might have different outcomes. Therefore, future investigations should include the collection of data from multiple centres in order to limit potential selection bias and achieve true randomization as well as being more applicable to other populations.

## Conclusions

In conclusion, patients with multiple sites of metastasis have a 2.5-fold higher risk of death compared to those with bone metastasis in terms of OS and MS. Moreover, CNS metastasis is also a predictor of a worse OS compared to bone metastasis. There was no difference in DRFS regarding the different solid organs or according to type of metastasis. Further prospective randomized controlled studies are needed in TNBC patients presenting metastasis to assess different therapeutic modalities and molecular biomarkers.

## Supporting information

S1 Checklist STROBE statement—checklist of items that should be included in reports of observational studies. (DOCX)

S1 Data Data set- survival according to the site of metastasis in triple-negative breast cancer patients: The Peruvian experience. (XLSX)
